# A Masi-Entropy Image Thresholding Based on Long-Range Correlation

**DOI:** 10.3390/e27121203

**Published:** 2025-11-27

**Authors:** Perfilino Eugênio Ferreira Júnior, Vinícius Moreira Mello, Enzo P. Silva Ribeiro, Gilson Antonio Giraldi

**Affiliations:** 1 Department of Mathematics, Federal University of Bahia, Salvador 40170-110, BA, Brazil; perfeuge@ufba.br (P.E.F.J.); vinicius.moreira.mello@gmail.com (V.M.M.); enzo.ribeiro@ufba.br (E.P.S.R.); 2 Coordination of Mathematical and Computational Methods, National Laboratory for Scientific Computing, Petrópolis 25651-075, RJ, Brazil

**Keywords:** image thresholding, entropy, local long-range correlation, infrared images

## Abstract

Entropy-based image thresholding is one of the most widely used segmentation techniques in image processing. The Tsallis and Masi entropies are information measures that can capture long-range interactions in various physical systems. On the other hand, Shannon entropy is more appropriate for short-range correlations. In this paper, we have improved a thresholding technique based on Tsallis and Shannon formulas by using Masi entropy. Specifically, we replace the Tsallis information measure with Masi’s one, obtaining better results than the original methodology. As the proposed method depends on an entropic parameter, we designed a thresholding algorithm that incorporates a simulated annealing procedure for parameter optimization. Then, we compared our results with thresholding methods that use just Masi (or Tsallis), or a combination of them, Shannon, Sine, and Hill entropies. The comparison is enriched with a kernel version of a support vector machine, as well as a discussion of our proposal in relation to deep learning approaches. Quantitative measures of segmentation accuracy demonstrated the superior performance of our method in infrared, nondestructive testing (NDT), as well as RGB images from the BSDS500 dataset.

## 1. Introduction

Image segmentation is a mid-level processing technique used for image analysis. Such a method consists of splitting an image into several disjointed parts by grouping the pixels to form homogeneous regions regarding pixel features like intensities, textures, heat signatures, among other characteristics. The regions formed should be visually distinct, and their homogeneity should be based on correlations among image pixels. Errors in the segmentation could be caused by effects of illumination, shadowing, noise, partial occlusion, and subtle object-to-background changes.

One of the most basic and well-known ways to segment grayscale images is thresholding, which works by selecting one or more gray levels of the image, called thresholds, to separate objects from their background. When the objective is to separate the object region (foreground) from the background through only one gray level, the method is called bi-level thresholding [1,2,3,4,5,6,7,8]. This form of region segmentation results in a binary image, in which each region is either white or black. Otherwise, if the separation of the regions of interest from the background depends on different gray tones, the process is called multilevel thresholding [9,10,11,12,13,14,15,16].

The focus of this work is bi-level image thresholding. Entropy-based approaches constitute the most common techniques in this area. Basically, thresholding is implemented through an optimization technique that consists of selecting a threshold that maximizes the entropy of the segmented image. For instance, Kapur et al. maximize the Shannon entropy of segmented classes to obtain the optimal threshold values [17]. Suboptimal results of image thresholding can also be considered to deal with the time-consuming computation involved. In this line, the work [8] proposed to segment infrared (IR) images based on the maximum entropy of 2D histograms and the PSO algorithm. Other works on the maximum entropy method proposed over the years have been based on Rényi’s [18], Tsallis [3], and Kaniadakis entropies [19].

IR images have a lot of low-frequency information, and the experimental tests have shown that some classical thresholding methods [5,6,7] are not efficient in this case. IR imaging is very useful in the military field for detecting objects with strong heat signatures, such as equipment and troop motions [20,21,22]. In addition, we can mention medical image applications [23], power equipment fault detection [24], and pedestrian detection on a scene [25].

Pixel intensities maintain a local long-range correlation in the neighborhood of the pixels in regions of an image. This fact is explored in [5], which is based on the long-range correlation between gray levels of an IR image and Tsallis entropy. In this case, the long-range interactions between the pixels are captured by the entropic parameter *q* inherent to the Tsallis entropy formula.

Another entropy from the context of thermodynamic science tested on IR images is the Masi one [6] that arises from the analysis between Rényi and Tsallis entropies [26]. These entropies cannot simultaneously manipulate long-range correlation, long-term memory, and fractal behavior [6]. Like Tsallis, Masi entropy is nonextensive, with a degree of nonextensivity measured by a parameter *r*.

The image histogram is a fundamental source of information for entropy-based methods [27,28]. In general, the appearance of a histogram is not that of two symmetrical portions with respect to an axis (threshold), which imposes difficulties on the thresholding techniques. To address this issue, ref. [29] presented a model that incorporates the Gumbel distribution to improve thresholding via cross-entropy in skewed histograms. In the same line, ref. [30] presents a method for automatic thresholding that allows selecting reasonable thresholds for images with unimodal, bimodal, multimodal, and peakless histograms.

The basic hypothesis for entropy-based bi-level thresholding methods is that the object and background regions are independent of each other. Recently, a method based on breaking this paradigm was proposed in [31], through the Tsallis entropy correlation.

In this work, we propose a new thresholding method based on long-range correlation and Masi entropy. Specifically, inspired by the methodology presented in [5], the novel technique computes the threshold through a max−min optimization problem that involves the Masi and Shannon entropies.

The parameter selection of nonextensive entropies requires some effort on the part of the user. Optimization techniques and empirical methods have been extensively researched on this subject [4,32,33]. However, nothing good enough to obtain an optimal parameter that provides better thresholding has yet emerged in the literature. In our case, besides the selection by the user himself, we have provided a thresholding algorithm that includes a procedure based on the simulated annealing technique to automatically seek the near-optimum value of the entropic parameter *r*.

The experiments are concentrated on IR, nondestructive testing (NDT) images, as well as a subset of the colored images taken from the BSDS5000 dataset [34]. The main goals are to demonstrate the generalization capabilities of our approach and to allow the comparison with competing works. The results proved to be very competitive in relation to those of [3,5,6], with very low error measures. The former is the baseline for our proposal, while [6] applies only Masi, and [3] uses only Tsallis entropy. These facts have motivated the comparison between these state-of-the-art approaches and our proposal. In addition, we have used more recent techniques published in [35,36,37,38], as well as a support vector machine (SVM) implementation, from the Scikit-Learn library, to show how the proposed method fares versus a traditional learning-based approach. The efficiency of our proposal is assessed through measures of segmentation accuracy, demonstrating the superior performance of our method in infrared, nondestructive testing (NDT), and RGB images from the BSDS500 dataset. We also discuss aspects of our proposal in comparison to deep learning.

The organization of this paper is as follows: Section 2 discusses works related to our proposal. Section 3 presents the entropy functions investigated here. In Section 4, our method is presented. Then, Section 5 presents a theoretical analysis based on graph elements and the Principle of Maximum Entropy (PME). Section 6 shows the obtained computational results, and Section 7 discusses the main findings and some issues. Additionally, in Appendix A, a variation of our thresholding algorithm is presented to support ablation analysis.

## 2. Related Work

Nowadays, image segmentation approaches can be divided into two classes: traditional and deep learning methods. The former is composed of techniques based on edge extraction, fuzzy and morphological concepts, region representation, partial differential equations (PDEs), graph formulations, as well as stochastic and thresholding approaches [39]. On the other hand, deep learning methods rely on the universality of neural network computing, large annotated databases, theoretical and hardware developments that allow the training of deep architectures in feasible times [40]. Hence, despite the outstanding results obtained by deep neural networks in segmentation tasks, such performance is only possible if there is sufficient data for training, with proper diversity and representativeness, or if we can design appropriate architectures and/or methodologies for transfer learning, data augmentation, and data imbalance, which are tasks with their own issues (see [41,42] and ([43], Section 2.2)).

Thresholding methodologies achieve competitive results even if a small dataset is accessible for testing [1]. These methods do not depend on time-consuming training stages, like deep learning and some stochastic approaches. The parameter setting is simpler than edge extraction (like active contour models) and graph formulations (like graph cuts). Its formulation is simple and intuitive, allowing the development of algorithms with complexity lower than PDE-based methods, especially in the bi-level case. Those observations support the research in thresholding methods nowadays, as we have noticed in recent works for segmentation of thermograms [44], medical imaging [45], satellite images [46], color images [47], among others [48,49].

In [1], thresholding methods are separated into six groups depending on whether they are based on features related to the histogram shape, object attributes, stochastic correlation between pixels, local image characteristics, clustering algorithms, or entropy concepts. The latter class is derived inside information theory methodologies that are grounded on statistical mechanics elements [50]. This viewpoint started in [2] by applying the Shannon entropy and the concept of anisotropy coefficient. On the other hand, if we consider the entropy as the measure of the information contained in the image, then the best threshold is the one that maximizes the entropy of the result. This line is followed by [17] that seeks a threshold *t* that partitions the histogram into two regions that maximize the Shannon entropy of the corresponding joint probability distribution. However, such a proposal may fail if applied to distinct images with identical histograms, as pointed out in [18]. One direction to address this issue is the application of more general entropic concepts that include new parameters that can be customized to fit the application requirements.

That is the case of Renyi’s entropy used in [18] for bi-level thresholding, which uses an approach analogous to the one presented in [17] but replacing Shannon entropy with Renyi’s one. Shannon and Renyi entropies characterize systems known as extensive ones [51]. On the other hand, some nonextensive systems follow another entropy formula proposed by Tsallis in [52].

Tsallis entropy includes the entropic parameter q∈R and we can show that, in the limit q→1, it equals the Shannon information measure. Tsallis entropy is the foundation of a new formalism in statistical mechanics where the level of nonextensivity of a physical system is quantified by the parameter *q* [53]. The work [3] was the first one to use the Tsallis entropy formalism for bi-level thresholding. Then, multilevel segmentation [9] approaches have been developed using Tsallis entropy with applications in medical imaging [45], multispectral image analysis [54], among others.

In this scenario, an important point is the distinction between long-range and short-range correlations. Specifically, image fields characterized by long-range correlations are more efficiently represented using Tsallis entropy, while Shannon entropy models short-range correlations [3,5].

Such a distinction raised the issue about the utilization of another entropy measure that is more flexible with respect to long-range correlation events but preserves the Shannon entropy characteristics. The Masi entropy, used in [6], is a choice in this direction that is dependent on the entropic parameter, *r* such that the limit r→1 recovers the Shannon measure. These facts have motivated our proposal that puts together Masi and Shannon entropies in a new thresholding approach. Our work is inspired by [5], which applies a max−min optimization problem that involves Tsallis and Shannon information measures.

## 3. Entropy Functions

Entropy is the measure of disorder in physical systems or the measure of the amount of information that may be needed to specify the full microstates of the system. In 1948, Shannon added entropy to information theory, and his approach measures the uncertainty associated with a random variable or the amount of information produced by a process [55] as

(1) $$S\left(X\right)=-\sum_{i=1}^{n}p_{i}\log p_{i},$$

where *X* is a random variable that can take values $\left\{x_{1},\ldots ,x_{n}\right\}$, and $p_{i}=p\left(x_{i}\right)$ is the corresponding probability of $x_{i}$. Equation (1) is the Shannon entropy that describes systems that obey the following additive property: Let *A* and *B* be two random variables associated with independent statistical subsystems of a physical system. Then,

(2) S(A+B)=S(A)+S(B).

Systems of this kind are called extensive systems. A certain class of physical systems, which entail long-range interactions, long-time memory, and fractal-type structures, indicated the need for an extension. Tsallis entropy [52] extends its applications to so-called nonextensive systems using an adjustable parameter *q*. Tsallis entropy can explain a complex system class, such as long-range and long-memory interactions. It can be expressed as

(3) $$S_{q}\left(X\right)=\frac{1}{1-q}\left(\sum_{i=1}^{n}p_{i}^{q}-1\right),$$

where *X* is a random variable, q∈R and $S_{q}\left(X\right)$ converge to S(X) in the limit q→1. The function $S_{q}\left(X\right)$ has the following pseudo-additivity property for q≠1:

(4) $$S_{q}\left(A+B\right)=S_{q}\left(A\right)+S_{q}\left(B\right)+\left(1-q\right)S_{q}\left(A\right)S_{q}\left(B\right),$$

where *A* and *B* are independent subsystems of a physical system. In 2005, Masi proposed a new entropy that combines the nonextensivity of Tsallis entropy and the additivity of Rényi’s entropy [26], namely

(5) $$S_{r}\left(X\right)=\frac{1}{1-r}\log\left[1-\left(1-r\right)\sum_{i=1}^{n}p_{i}\log p_{i}\right],$$

where r∈R(r>0 and r≠1). Moreover, $S_{r}$ satisfies

(6) $$S_{r}\left(A+B\right)=S_{r}\left(A\right)+S_{r}\left(B\right),$$

with *A* and *B* as before. The parameters *q* and *r* can be viewed as measures for the degree of nonextensivity that exists in the system for the Tsallis and Masi entropies, respectively.

### Entropy Functions and Image Thresholding

For the image thresholding context, we will consider an image with *n* gray levels, and let $p_{1},p_{2},\ldots ,p_{n}$ be the probability distribution of the levels. Here, two probability distributions can be derived from the original distribution, one for the background (class *A*) and the other for the object (class *B*). Their probabilities can be given by

(7) $$A:\frac{p_{1}}{P_{A}},\frac{p_{2}}{P_{A}},\ldots ,\frac{p_{t}}{P_{A}},\;B:\frac{p_{t+1}}{P_{B}},\frac{p_{t+2}}{P_{B}},\ldots ,\frac{p_{n}}{P_{B}},$$

where $P_{A}=\sum_{i=1}^{t}p_{i}$ and $P_{B}=\sum_{i=t+1}^{n}p_{i}$.

In this case, Albuquerque’s method obtains an optimal threshold by maximizing the information measure between the two classes, where the objective function $S_{q}^{A+B}=S_{q}^{A}+S_{q}^{B}+\left(1-q\right)S_{q}^{A}S_{q}^{B}$ is parametrically dependent upon the threshold value *t* that separates the foreground and background. Hence, Albuquerque’s solution is given by

(8) $$t_{opt}=argmax\left[S_{q}^{A+B}\left(t\right)\right].$$

The nonextensive parameter *q* represents the strength of the long-range correlation. Equation (4) indicates that there is a global correlation not only in the areas of foreground and background but also between them. Moreover, the strength of the global correlation is described by the same value *q*.

Regarding Masi entropy applied in Nie et al.’s method [6], although this entropy is nonextensive, it is also additive. The parameter *r* establishes the degree of nonextensivity and the strength of the long-range correlation property. The long-range correlation could be indicated by the Equation (6) whose optimum,

(9) $$t_{opt}=argmax\left[S_{r}^{A+B}\left(t\right)\right],$$

gives the threshold value in [6].

In practice, the global long-range correlation indicated by Equation (4) does not apply for IR images since the thresholding results only show local long-range correlation [5] in this case. Thus, the optimal value found by the maximization process is not sensitive to small variations of the nonextensive parameter *q*, as reported in [5]. In that reference, the authors argue that it would be inappropriate for these types of images to say that there is a global long-range correlation. The long-range correlation would be weaker in the background of some images in the context of Tsallis entropy. Thus, instead of simply maximizing the sum of the entropies of object and background, they decided to maximize both the Shannon entropy on the background of the image and Tsallis on the object. However, it is hard to obtain the absolute maxima of them with a single threshold unless their thresholds are equal. For this reason, the solution involved a trade-off that is addressed in [5] as the optimization problem:

(10) $$t_{opt}=argmax\left\{min\;\left[S^{A}\left(t\right),S_{q}^{B}\left(t\right)\right]\right\},$$

where $S^{A}$ is the Shannon entropy calculated on the background of the image and $S_{q}^{B}$ is the Tsallis entropy calculated on the object. Some tested images apparently had a stronger long-range correlation in the background of the image. To mitigate this effect, Lin & Ou [5] also proposed an alternative way for the trade-off, interleaving the entropies:

(11) $$t_{opt}=argmax\left\{min\;\left[S_{q}^{A}\left(t\right),S^{B}\left(t\right)\right]\right\}$$

where $S_{q}^{A}$ is the Tsallis entropy calculated on the background of the image and $S^{B}$ is the Shannon entropy calculated on the object.

## 4. Proposed Method

Our proposal is to combine the Masi and Shannon entropies in a trade-off similar to that used by Lin & Ou in Equations (10) and (11). Two facts motivated the current proposal involving Masi entropy. First, its image thresholding results do not present a weak correlation in the background of images for the appropriate parameter *r*. Therefore, Masi entropy could be thought of as a method based on global long-range correlation. Second, the optimal value found in the maximization process of Masi entropy is very sensitive to the variation of the nonextensive parameter *r*. Thus, we propose a trade-off as follows:

(12) $$\mathrm{Proposal}\;1:\;\;t_{opt}=argmax\left\{min\;\left[S^{A}\left(t\right),S_{r}^{B}\left(t\right)\right]\right\}$$

where $S^{A}$ is the Shannon entropy calculated on the background of the image and $S_{r}^{B}$ is the Masi entropy calculated on the object. When there are distributions of different gray levels in the image, we can propose an alternative way as in Equation (11).

(13) $$\mathrm{Proposal}\;2:\;\;t_{opt}=argmax\left\{min\;\left[S_{r}^{A}\left(t\right),S^{B}\left(t\right)\right]\right\}$$

where $S_{r}^{A}$ is the Masi entropy calculated on the background of the image and $S^{B}$ is the Shannon entropy calculated on the object. This would be the case of having a weaker long-range correlation in the background of the image, for example.

The selection of *r* value can be executed by trial-and-error tests like performed in [3,5,6] to set *q* and *r* values. Although some works have been developed to automatically set entropic parameters [4,56,57], this subject remains an open issue in image processing. In our case, we uniformly sample a set of values over a predefined interval and select the one that best approximates the target segmentation, according to problem (9), to refine the search. The obtained value $r=r_{★}$ is used to initialize the search for the best *r* for problem (12). Additionally, we offer a thresholding algorithm that includes a SA technique for entropic parameter optimization, to improve reproducibility and avoid ad hoc procedures for seeking the near-optimum *r* for our proposal.

The alternative form is used when a thresholding result close to the optimal value is not achieved by Equation (12). Then, we seek another *r* value to execute the optimization process given by Equation (13), instead of Equation (12). Experimentally, in all cases, it was possible to obtain a near-optimum solution through the ability to capture the long-range correlation properties among the pixels due to Masi entropy. In the following section, we report the results and offer quantitative comparisons with the other methods.

## 5. Theoretical Analysis

This section offers a justification for the proposed criterion in expressions (12)–(13) and uses the Principle of Maximum Entropy (PME) to discuss the long-range and short-range correlations in Tsallis, Shannon, and Masi entropies.

### 5.1. Justification by Isoperimetric Theory

It this section we follow [7,38] and apply graph elements to give a mathematical justification for Proposals 1 and 2 in Section 4. Thus, we start by modeling a grayscale image as a weighted undirected graph $G=\left(V,E\right)$, with *V* being the set of pixels and E⊂V×V being a set of edges $e_{ij}=\left(v_{i},v_{j}\right)\in V\times V$ linking adjacent pixels $v_{i}$ and $v_{j}$ in *V*. Furthermore, we define a function $w:E⟶R_{+}$ that calculates normalized weights:

(14) $$w_{ij}=\frac{\exp\left(-\beta {\left(I\left(v_{i}\right)-I\left(v_{j}\right)\right)}^{2}\right)}{\sum_{E}\exp\left(-\beta {\left(I\left(v_{m}\right)-I\left(v_{n}\right)\right)}^{2}\right)},$$

where $I:V⟶\left\{0,1,2,\ldots ,L\right\}$ represents gray level function. Hence, we define the following:

- Degree of $v_{i}\in V$: $d_{i}=\sum w_{ij},$$\forall e_{ij}\in E$.
- Complement of *S*: $\bar{S}=V-S$.
- Volume of *S*:
  (15) $$d_{S}=\sum_{v_{i}\in S}d_{i},$$

Given S⊂V, the boundary of *S* is represented as $\partial S=\left\{e_{ij}|\;v_{i}\in S\;and\;v_{j}\in \bar{S}\right\}$. Then, we can define

(16) $$\mathrm{cut}\left(S,\bar{S}\right)=\sum_{e_{ij}\in \partial S}w_{ij}.$$

In the thresholding segmentation theory, each set *S* is specified by a threshold *t* and, consequently, $S=S\left(t\right)$. With those elements, to each set $S\left(t\right)\subset V$, we can define the isoperimetric ratio as follows [58]:

(17) $$h\left(S\left(t\right)\right)=\frac{\mathrm{cut}\left(S\left(t\right),\bar{S}\left(t\right)\right)}{\min\left(d_{S\left(t\right)},d_{\bar{S}\left(t\right)}\right)}.$$

Now, a global graph feature named isoperimetric constant is computed as follows [58]:

(18) $$h_{G}=\min_{0\leq t\leq L}\frac{\mathrm{cut}\left(S\left(t\right),\bar{S}\left(t\right)\right)}{\min\left(d_{S\left(t\right)},d_{\bar{S}\left(t\right)}\right)}.$$

Expression (18) is applied as a segmentation criterion in [58]. The justification behind such a criterion is that $\mathrm{cut}\left(S,\bar{S}\right)$ decreases when considering regions *S* and $\bar{S}$ with high contrast between each other. Moreover, some degree of homogeneity is expected in the interior of *S* (or $\bar{S}$), which increases the value of $d_{S}$ (or $d_{\bar{S}}$). By considering $\min\left(d_{S},d_{\bar{S}}\right)$ in the denominator, there is a preference for uniform-intensity regions instead of larger areas. Homogeneous-intensity regions decrease the Masi and Shannon entropies since their maximum occurs for regions with a uniform-intensity histogram. Consequently, we can replace $d_{S}$ and $d_{\bar{S}}$ to $S^{A}\left(t\right)$ and $S_{r}^{B}\left(t\right)$ in expression (18) to obtain

(19) $$h_{G}=\min_{0\leq t\leq L}\frac{\mathrm{cut}\left(S\left(t\right),\bar{S}\left(t\right)\right)}{\min\left(S^{A}\left(t\right),S_{r}^{B}\left(t\right)\right)}.$$

However, the weights $w_{ij}$ computed through Equation (14) are normalized, resulting in

(20) $$h_{G}=\min_{0\leq t\leq L}\frac{\mathrm{cut}\left(S,\bar{S}\right)}{\min\left(S^{A}\left(t\right),S_{r}^{B}\left(t\right)\right)}\leq \min_{0\leq t\leq L}\frac{1}{\min\left(S^{A}\left(t\right),S_{r}^{B}\left(t\right)\right)},$$

which renders

(21) $$t_{opt}=\arg\max_{0\leq t\leq L}\min\left(S^{A}\left(t\right),S_{r}^{B}\left(t\right)\right),$$

which is the proposed criterion in Equation (12). Analogously, we obtain Equation (13) by replacing $d_{S}$ and $d_{\bar{S}}$ to $S_{r}^{A}\left(t\right)$ and $S^{B}\left(t\right)$ in expression (18).

### 5.2. Long-Range Versus Short-Range Correlations

In this section, we follow [59] to formalize the stationary short-range correlation by an exponential function:

(22) $$S\left(x,y;\theta ,\sigma \right)=\frac{1}{2\theta }\exp\left(-\frac{∥x-y∥}{\sigma }\right),\;\theta ,\sigma >0,$$

while the stationary long-range correlation will be described through the power-law function:

(23) $$L\left(x,y;\tau ,\mu ,\gamma \right)=\frac{\tau^{-1}}{{\left(1+∥x-y∥/\gamma \right)}^{\mu }},\;\tau ,\mu >0.$$

**Figure 1:**
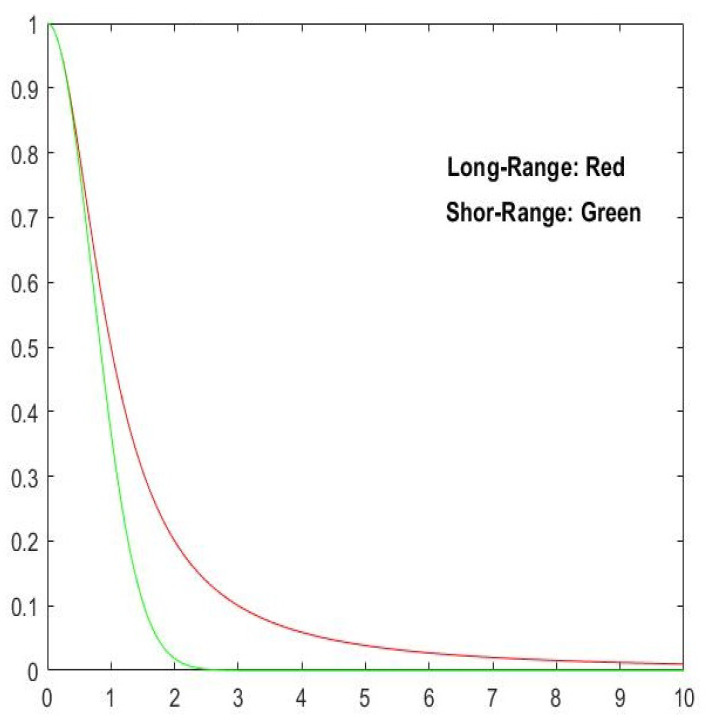
Plots of functions (22) and (23) showing short-range correlation in green and long-range correlation in red, respectively (Source [38]).

Firstly, if we perform the substitution $∥x-y∥⟶d$ in expressions (22)–(23) and set θ=0.5, σ=1.0, and τ=μ=1.0, we obtain the plot in Figure 1:

We notice that for both models, the correlation decreases with respect to the distance *d* but function $S\left(x,y;0.5,1.0\right)$ decays faster than its counterpart. Expressions (22)–(23) are inspired in Shannon (*S*) and Tsallis ($S_{q}$) entropies, respectively, as defined by expressions (1) and (3).

The second-order statistics associated with the information measures (1)–(3) are expressed by

(24) $$\sum_{i=1}^{W}p_{i}=1,$$

(25) $$\frac{\sum_{i=1}^{W}e_{i}p_{i}^{q}}{\sum_{i=1}^{W}p_{i}^{q}}=U_{q},$$

(26) $$\frac{\sum_{i=1}^{W}{\left(e_{i}-U_{q}\right)}^{2}p_{i}^{q}}{\sum_{i=1}^{W}p_{i}^{q}}=V_{q},$$

where equation (24) is necessary for $p_{i}$ to be the probability associated to the state $e_{i}$, for q=1 expressions (25)–(26) recover the regular mean and variance quantities.

The Tsallis entropy offers a new formalism in which the real parameter *q* quantifies the level of nonextensivity of a physical system [53]. In particular, a general Principle of Maximum Entropy (PME) has been considered to find out distributions to describe such systems. In this PME, the goal is to seek the maximum of $S_{q}$ subjected to constraints (24) and (26), which gives the solution:

(27) $$p_{j}=\frac{{\left[1+\frac{\left(q-1\right)}{k}\beta \left(\frac{{\left(e_{j}-U_{q}\right)}^{2}-V_{q}}{\sum_{i=1}^{W}p_{i}^{q}}\right)\right]}^{\frac{1}{1-q}}}{\sum_{j=1}^{W}{\left[1+\frac{\left(q-1\right)}{k}\beta \left(\frac{{\left(e_{j}-U_{q}\right)}^{2}-V_{q}}{\sum_{i=1}^{W}p_{i}^{q}}\right)\right]}^{\frac{1}{1-q}}},$$

where β is a Lagrange multiplier and the denominator is just a normalization factor in order to fit constraint (24). Although expression (27) is not practical since the right-hand side also depends on the unknowns $p_{j},$ it is usefull from theoretical viewpoint. Specifically, by comparing this expression with function (23) with the substitution $∥x-y∥⟶d$, we notice that if we set

$$d=\left(q-1\right)\left(\frac{{\left(e_{j}-U_{q}\right)}^{2}-V_{q}}{\sum_{i=1}^{W}p_{i}^{q}}\right),$$

$$\gamma =\frac{k}{\beta },$$

$$\tau =\sum_{j=1}^{W}{\left[1+\frac{\left(q-1\right)}{k}\beta \left(\frac{{\left(e_{j}-U_{q}\right)}^{2}-V_{q}}{\sum_{i=1}^{W}p_{i}^{q}}\right)\right]}^{\left(\frac{1}{1-q}\right)},$$

$$\mu =-\left(\frac{1}{1-q}\right),$$

then we recognize the power-law kernel in both expressions (23)–(27), indicating that Tsallis entropy is more appropriate for model long-range correlation. On the other hand, an analogous PME can be formalized using the Shannon entropy (1). In this case, we must set q=1 in the expression (25) and the obtained result is

(28) $$p_{j}=\frac{\exp\left(-\beta {\left(e_{j}-U_{1}\right)}^{2}\right)}{\left(\sum_{j=1}^{W}\exp\left(-\beta {\left(e_{j}-U_{1}\right)}^{2}\right)\right)},$$

where β is the Lagrange multiplier again. In this case, the comparison with the stationary short-range model (Equation (22)) shows that the exponential function dominates both expressions.

On the other hand, when considering the PME for Masi entropy, we obtain

(29) $$p_{j}=\frac{\exp\left[-\beta r\left(1+(1-r)S\right)p_{j}^{r-1}\left(\frac{{\left(e_{j}-U_{r}\right)}^{2}-V_{r}}{\sum_{i=1}^{W}p_{i}^{r}}\right)-S\right]}{\sum_{j=1}^{W}\exp\left[-\beta r\left(1+(1-r)S\right)p_{j}^{r-1}\left(\frac{{\left(e_{j}-U_{r}\right)}^{2}-V_{r}}{\sum_{i=1}^{W}p_{i}^{r}}\right)-S\right]},$$

which shows a more complex dependency that includes the Shannon entropy (*S*) besides the power-law $p_{j}^{r-1}$. Hence, expression (29) shows that Masi entropy is more flexible regarding the trade-off long-range versus short-range correlation events being more appropriate for a wide range of images, as demonstrated in the computational experiments.

## 6. Experimental Results

In this section, the computational experiments are presented to demonstrate the capabilities of our proposal for segmentation tasks. Firstly, the experiments focus on IR and nondestructive testing (NDT) images to allow the comparison with the baseline [5], as well as [6,17] for ablation analysis, since [6] applies only Masi and [17] applies only Shannon entropy. Additionally, comparisons with [3] that uses only Tsallis is provided since we replace the Tsallis entropy with Masi’s one in our approach.

The parameterization of our proposal is also a point focused in the computational experiments since the results of Section 6.1 and Section 6.2 are obtained with ad hoc strategies. In this way, in Section 6.3, we present an algorithm that summarizes our proposal and includes a simulated annealing strategy to select the *r* value. With this algorithm, parameter selection and sensitivity analysis are discussed.

Section 6.4 delves into the ablation study by isolating the effect of Masi entropy in our proposal. Then, to show that our segmentation approach can be successfully applied to other types of images besides IR and NDT ones, experiments involving BSDS500 images are presented in Section 6.5. Additionally, a comparison with more recent entropy-based approaches is presented, and a support vector machine (SVM) is employed to demonstrate how the proposed method performs in comparison to learning-based approaches.

The set of NDT images used is composed of grayscale images with resolution 256×256 pixels. With respect to the IR images, they are also grayscale ones, but the resolution varies from 160×182 to 320×240 pixels. Both datasets can be acquired from the Google Drive (https://drive.google.com/file/d/1LzQNCjK4YeTzqGf-hYQ7Q_osi2Ery9m8/view?usp=sharing, (accessed on 15 September 2025)) and were assembled from freely available images on the Web, which were also used elsewhere [5,6,7,38].

**Figure 2:**
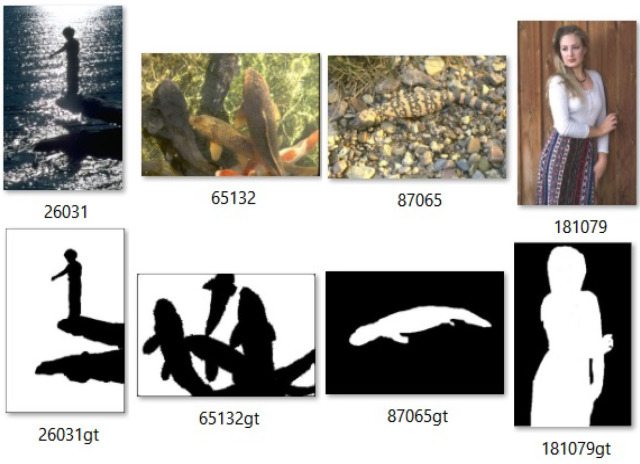
Some images of BSDS500-50 dataset with ground truth.

The BSDS500 database [34] is composed of RGB images with resolution 481×321 pixels selected from the Corel image database using the following criterion: *natural scenes that contain at least one discernible object.* Besides the RGB images, the BSDS500 database gives the corresponding segmentation, given by the delineation of the target boundary of the objects by humans. Such ground truth is not suitable for our purposes, which require pixel-wise classification between foreground and background. Hence, we took 50 images and pre-processed their segmentations to generate the ground truth with the desired characteristics for our application. The obtained dataset is named BSDS500-50 throughout this text. Figure 2 presents some samples of BSDS500-50 together with their ground truth.

To infer the performance of our method, we adopted four quantitative measures. One of them is the misclassification error (ME) measure [1]. It can be written as

(30) $$ME=1-\frac{\left|B_{GT}\cap B_{T}\right|+\left|F_{GT}\cap F_{T}\right|}{\left|B_{GT}\right|+\left|F_{GT}\right|}$$

where $B_{GT}$ and $B_{T}$ are the pixels of background in the ground-truth image and thresholded image, $F_{GT}$ and $F_{T}$ are the pixels of foreground in the ground-truth image and thresholded image, and |∘| is the cardinality of a set. The ME varies from 0 for a perfectly segmented image to 1 for a totally wrong binarized image. Another measure is the Jaccard similarity (JS), used in [20,60], which can be written as

(31) $$JS\left(F_{GT},F_{T}\right)=\frac{\left|F_{GT}\cap F_{T}\right|}{\left|F_{GT}\cup F_{T}\right|}.$$

Differently from Equation (30), the correct segmentation obtains JS=100%. The third measure is the relative foreground area error (RAE) [61] that represents the accuracy and completeness of the segmented foreground. It can be expressed as

(32) $$RAE=\frac{\left|\;\left|F_{GT}\right|-\left|F_{T}\right|\;\right|}{max(\left|F_{GT}\right|,\;\left|F_{T}\right|)}.$$

The lower the RAE value, the better the segmentation result. The fourth measure is F-measure [62,63] that is defined as the harmonic mean of precision and recall rate with weight α, which represents a compromise between under-segmentation and over-segmentation. Its value is provided by the expression

(33) $$F_{\alpha }=\frac{(1+\alpha )\cdot P\cdot R}{(\alpha \cdot P)+R},$$

where *P* and *R* are the precision and recall rates given by

$$P=\frac{\left|F_{GT}\cap F_{T}\right|}{\left|F_{T}\right|}\;and\;R=\frac{\left|F_{GT}\cap F_{T}\right|}{\left|F_{GT}\right|}.$$

Generally, larger values for *P* and *R* indicate better segmentation results. $F_{\mathrm{ff}}$ could be a balanced measure for segmentation results through a good choice of harmonic coefficient α. Usually, α is set to 0.5 for segmentation algorithms [62,63]. However, a segmentation that matches the ground truth obtains $F_{\alpha }=1$ regardless of the value of α.

In addition, we apply the uniformity measure (UM) to evaluate the segmentation when the ground truth is not available. Specifically, given regions $R_{1}$ and $R_{2}$ of the grayscale image *I*, defined by the segmented image, the UM is computed as follows [64]:

(34) $$UM\left(R_{1},R_{2}\right)=1-\frac{2\cdot \left(\sigma_{1}^{2}+\sigma_{2}^{2}\right)}{N\cdot M\cdot {\left(I_{max}-I_{min}\right)}^{2}},$$

where $\sigma_{i}^{2}$ is the variance with respect to the mean of region $R_{i}$, N,M are the image dimensions, and $I_{max},I_{min}$ denote the maximum and minimum pixel intensities in the image. Consequently, a higher UM value indicates lower intra-region variances.

The most detailed part of applying parameterized entropies is choosing the entropic parameters. For the experiments performed with Tsallis entropy, the values of *q* were obtained as follows:

1. We automatically searched, in the range [0.1,0.9] with increments of 0.1 units, for the best value of *q* that minimized the ME measure between the segmented image using just the Tsallis entropy (Equation (4)) and the ground-truth image.
2. From this, the parameter *q* found is used in the combination of the Tsallis and Shannon entropies (Equation (10)). This can be corroborated in all the Figures generated in the text.
3. If ME value is larger than 0.1 then the same procedure is repeated using Equation (11).

On the other hand, the entropic parameter r is somewhat more complicated to handle. The following steps were next performed in Section 6.1 and Section 6.2:

- The variation range of *r*, including all types of images tested, was in the range [0.7,1.7], with increments of 0.1 units.
- As we vary *r* in Masi expression (9), we notice that the ME values could be improved by oversampling within the current range. For example, if the ME value decreases for *r* within the subrange [1.3,1.4], closer to the value 1.3, we then test the value r=1.32. If the ME value decreases, we establish a new subinterval around this point, say [1.312,1.329]. In such a range, we should find a value of $r_{*}$ that minimizes ME.
- Unlike the Tsallis entropy parameter, in general, the best value of $r_{*}$ for Masi entropy alone does not coincide with the best *r* value for the proposed max–min strategy in Equation (12). In the latter case, the same procedure is applied (items (a)–(b)), but now using Equation (12).

Items (a)–(c) above are ad hoc. A systematic algorithm will be presented in Section 6.3, based on simulated annealing (SA), to improve the generalization and reproducibility of our results.

### 6.1. Performance on IR Images

Table 1 shows thresholding values, as well as the number of misclassified pixels, ME, JS, RAE, and $F_{\alpha }$ values, for the images. Additionally, the results with lower ME and RAE values and higher JS and $F_{\alpha }$ values are marked in bold. We can observe in the last column of Table 1 that our proposal proved to be the most efficient, or tied for the best, among the experiments carried out.

**Figure 3:**
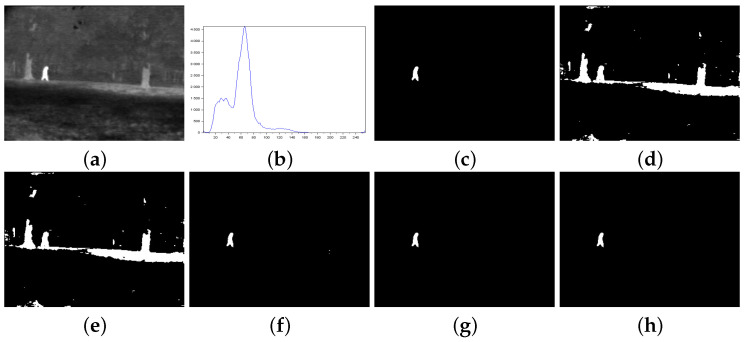
Thresholding results of 000280 image: (**a**) original, (**b**) Histogram, (**c**) ground-truth image, (**d**) Shannon method (threshold value T=91), (**e**) Albuquerque’s method (entropic parameter q=0.8 and T=91), (**f**) Lin & Ou’s method (q=0.8 and threshold threshold T=169), (**g**) Nie et al.’s method (entropic parameter r=1.35 and T=173), and (**h**) the proposed method (r=1.21 and threshold T=170).

**Figure 4:**
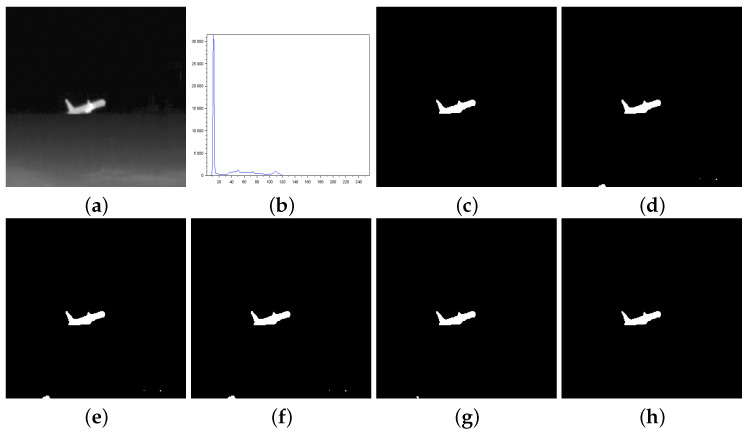
Thresholding results of Airplane image: (**a**) original, (**b**) Histogram, (**c**) ground-truth image, (**d**) Shannon method (threshold value T=117), (**e**) Albuquerque’s method (entropic parameter q=0.8 and threshold value T=117), (**f**) Lin & Ou’s method (q=0.8 and T=117), (**g**) Nie et al. method (entropic parameter r=1.22 and threshold value T=121), and (**h**) the proposed method (r=0.797 and T=123).

**Figure 5:**
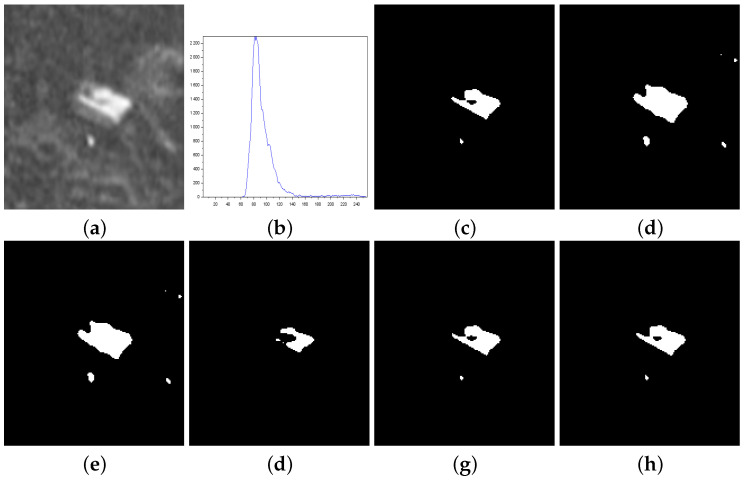
Thresholding results of Tank image: (**a**) original, (**b**) Histogram, (**c**) ground-truth image, (**d**) Shannon method (threshold value T=140), (**e**) Albuquerque’s method (entropic parameter q=0.9 and threshold value T=140), (**f**) Lin & Ou’s method (q=0.9 and T=220), (**g**) Nie et al.’s method (entropic parameter r=1.25 and T=194), and (**h**) the proposed method (r=0.99 and T=191).

**Figure 6:**
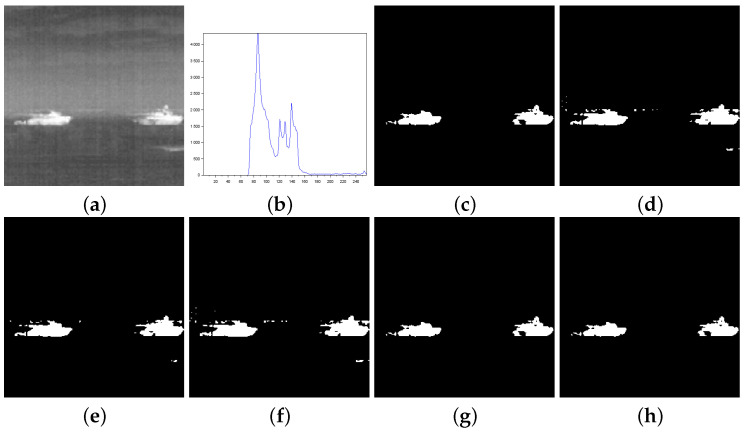
Thresholding results of Panzer image: (**a**) original, (**b**) Histogram, (**c**) ground-truth image, (**d**) Shannon method (threshold value T=154), (**e**) Albuquerque’s method (entropic parameter q=0.1 and threshold value T=157), (**f**) Lin & Ou’s method (q=0.1 and T=153), (**g**) Nie et al.’s method (entropic parameter r=1.231 and T=174), and (**h**) the proposed method (r=0.997 and T=174).

**Figure 7:**
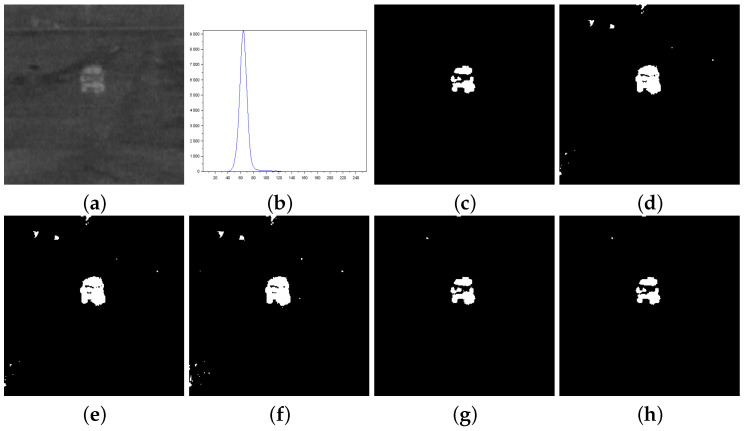
Thresholding results of Car image: (**a**) original, (**b**) Histogram, (**c**) ground-truth image, (**d**) Shannon method (T=81), (**e**) Albuquerque’s method (entropic parameter q=0.9 and threshold value T=81), (**f**) Lin & Ou’s method (q=0.9 and T=80), (**g**) Nie et al.’s method (entropic parameter r=1.28 and T=89), and (**h**) the proposed method (r=0.95 and T=90).

**Figure 8:**
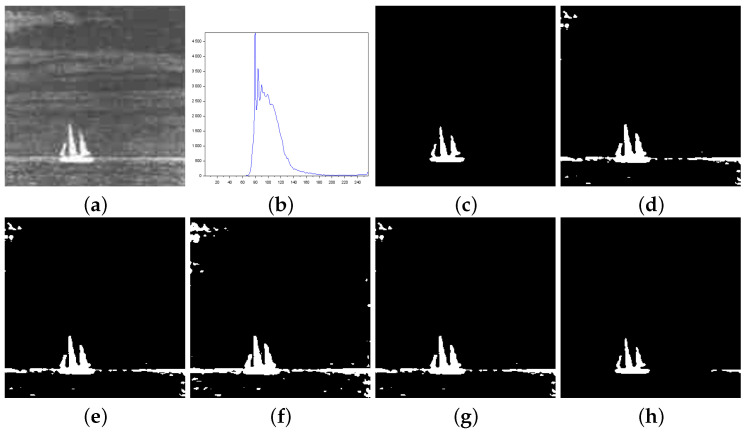
Thresholding results of Sailboat image: (**a**) original, (**b**) Histogram, (**c**) ground-truth image, (**d**) Shannon method ( threshold value T=155), (**e**) Albuquerque’s method (entropic parameter q=0.6 and T=155), (**f**) Lin & Ou’s method (q=0.6 and T=141), (**g**) Nie et al.’s method (entropic parameter r=0.9 and T=155), and (**h**) the proposed method (r=1.03 and T=193).

Figure 3, Figure 4, Figure 5, Figure 6, Figure 7 and Figure 8 show more details about the results generated with the reported image thresholding methods:

- Figure 3—000280 image: We notice that Lin & Ou’s method had an RAE equal to zero, which implies that the number of pixels in the foreground of the ground truth matched the number of pixels in the foreground of the thresholded image. However, visually, we can notice differences between the ground truth and the segmented image by Lin & Ou’s method. The other measures corroborate this observation, showing that the behavior of our method was slightly better than that of Lin & Ou’s as well as Nie et al.’s methods, whose results were similar. The proposed method exhibited the highest **JS** value for that image.
- Figure 4—Airplane image: Our proposal surpasses all the others obtaining JS (Equation (31)) greater than 99% and a $F_{\alpha }$ value close to 1. Albuquerque’s and Lin & Ou’s methods match up and show inferior results if compared to the second-best approach, that is, Nie et al.’s method in this case. The image obtained with Lin & Ou’s method was generated with the alternative form given by Equation (11). This should be an indication that the correlation in the region of background is most strongly captured by Tsallis entropy (see the sharp peak in the histogram in Figure 4b).
- Figure 5—Tank image: Our method overcomes all other techniques and provides more than 98% of Jaccard similarity (Equation (31)). This example shows a significant difference with respect to the misclassified pixels and JS value in relation to the second-best result, that of Nie et al. Albuquerque and Lin & Ou’s methods perform far from our technique for all the considered measures.
- Figure 6—Panzer image: Our method and Nie et al.’s approach achieve perfect segmentation. The number of misclassified pixels, ME, and RAE values are zero. The image obtained with Lin & Ou’s method was generated with the alternative form (11). However, both Albuquerque and Lin & Ou’s methods obtain segmentations far from the ground truth, as indicated by the values of the considered measures.
- Figure 7—Car image: Our result outperforms their counterparts. Again, the alternative form (11) for Lin & Ou’s method was used to generate the corresponding image.
- Figure 8—Sailboat image: Our results are much superior to other methods for all the measures considered. The image obtained with Lin & Ou’s method was generated with the alternative form (11).

The error measures reported in Table 1 show that our proposal is more efficient than competing techniques. The second-best technique, Nie et al.’s method, performs similarly as our approach only in the Panzer image, being inferior in the other cases. In addition, our proposal for image thresholding based on local long-range correlation and Masi entropy provides much better results than those based on Tsallis entropy. A range of *q* values used to generate images of Albuquerque’s and Lin & Ou’s methods was 0.1≤q≤0.9.

The ranges of *r* values used for Nie et al.’s method and ours were 0.9≤r<1.4 and 0.7<r<1.3 (r≠1), respectively. As observed in [5], the optimal value found by the Tsallis entropy maximization process is not sensitive to the small variations in *q*. Differently, Masi entropy maximization process is quite sensitive to variations in *r*, even small ones. This fact is relevant for establishing the step for sampling the above interval to set *r* in Equations (12) and (13).

**Figure 9:**
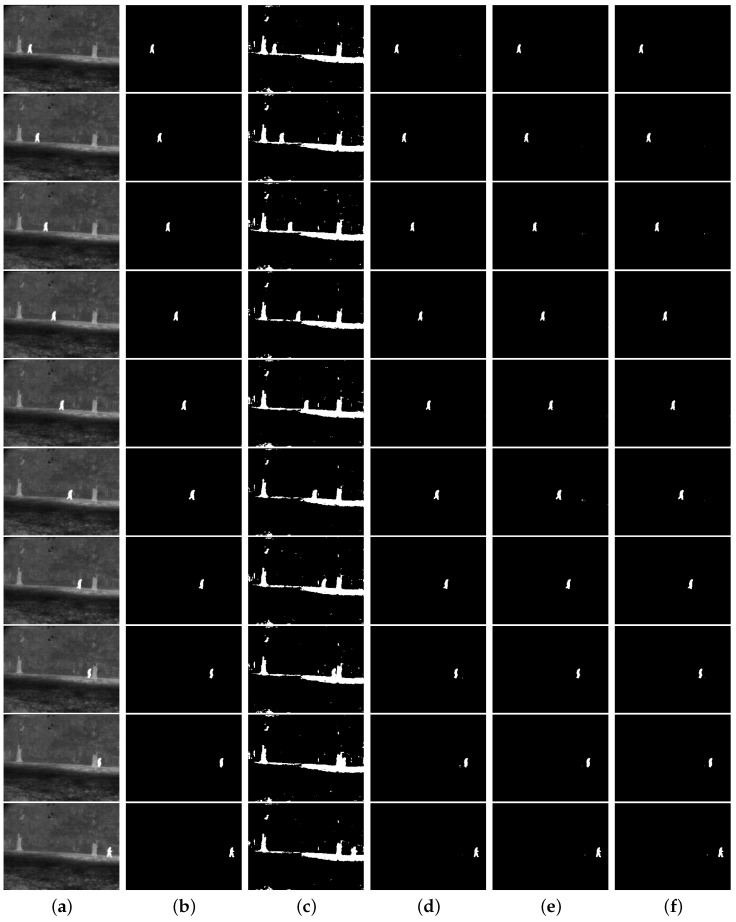
Infrared image sequence and their thresholding results: (**a**) original, (**b**) ground-truth image, (**c**) Albuquerque’s method (entropic parameter q=0.8), the thresholds from top to bottom: 91, 93, 91, 97, 90, 98, 93, 96, 94, 96, (**d**) Lin & Ou’s method (entropic parameter q=0.8), the thresholds from top to bottom: 169, 178, 185, 177, 179, 178, 179, 173, 175, 192, (**e**) Nie et al.’s method, the parameters *r* and thresholds from the top to bottom: (1.35,173), (1.341,177), (1.31,171), (1.34,176), (1.334,175), (1.32,170), (1.334,177), (1.35,176), (1.35,178), (1.32,182), (**f**) the proposed method, the parameters *r* and thresholds from top to bottom: (1.21,170), (1.19,174), (1.15,170), (1.205,177), (1.192,175), (1.19,174), (1.19,177), (1.21,175), (1.22,178), (1.17,181).

Figure 9 shows a test performed with an image sequence. Such images contain an object (a person) moving on the same background. As in Lin & Ou’s work [5], we assume that the strength of the long-range correlation of the images should be similar for Tsallis entropy. The authors judged that the correlation strength would be the same for that object on the images, which yields the same optimal *q* value. Thus, we kept *q* fixed at 0.8, the same value for image 000280 from Figure 3. For the Nie et al method and ours, the ranges of *r* values found were 1.3≤r<1.4 and 1.1≤r<1.3, respectively.

It can be seen from Table 1 that the Shannon entropy data for IR images are the same as those for Tsallis entropy, except for the Panzer image. Even so, the measurement values were relatively close in that case. A possible cause for this effect is that the Shannon entropy is obtained from the Tsallis entropy as *q* tends to 1, since in our experiments, we use q≥0.8. In fact, in general, the Tsallis entropy does not change much with variations in *q*. Therefore, given a value $q_{1}$, the Tsallis entropy would remain almost constant within a certain range around the $q_{1}$ value. Thus, for *q* close to 1, the values of the Tsallis and Shannon entropies can be nearly equal, leading to similar segmentation results. For this reason, the experiment with the image sequence in Figure 9 was not performed with Shannon entropy.

Table 2 shows the JS obtained for this experiment. Our approach achieves the best results or performs equally well as the best methods. Lin & Ou’s method ties with our approach in only one of the listed cases (000340 image). Nie et al.’s method ties with our method in three of the images in the sequence (000360, 000400, and 000440 images).

Table 3 shows the threshold values, misclassified pixels, and misclassification error (ME) for the segmentation of the infrared images (IR) in Figure 9. Data in Table 3 corroborate our approach as the best among those analyzed since it outperforms the competing methods or ties with the best-performing one. Specifically, Table 3 shows a tie between the proposed method and the results of Lin & Ou’s technique (000340 image) and four ties with the Nie et al. approach (000320, 000360, 000400, and 000440 images). This highlights the competitive potential of the proposed approach against Lin & Ou and Nie et al. methods, which are based on nonextensive entropies. In the 000320 image, the ME and misclassified pixel values for our method and those of Nie et al. are the same, given by 0.000143229 and 11, respectively. Despite this, Table 2 reports that our approach obtained a **JS** value of 94.93%, while Nie et al. obtained 94.91%. Hence, we notice that the difference between their **JS** values is 2 hundredths. This shows a slight advantage of our approach over Nie et al.’s method in this case.

### 6.2. Performance on NDT Images

**Figure 10:**
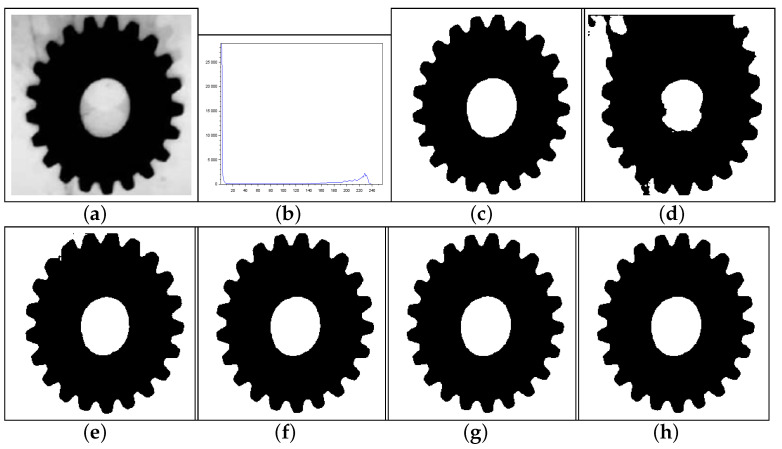
Thresholding results of Gear image: (**a**) original, (**b**) Histogram, (**c**) ground-truth image, (**d**) Shannon method (threshold value T=185), (**e**) Albuquerque’s method (entropic parameter q=0.1 and T=128), (**f**) Lin & Ou’s method (q=0.6 and T=87), (**g**) Nie et al.’s method (entropic parameter r=1.22 and T=75), and (**h**) the proposed method (r=1.7 and T=80).

**Figure 11:**
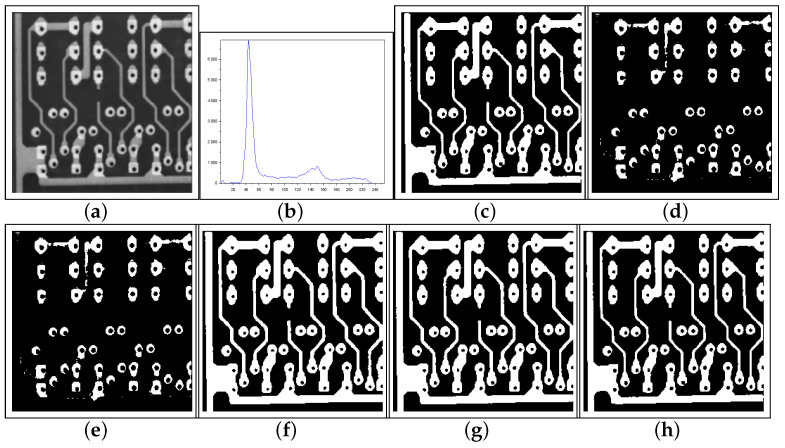
Thresholding results of Pcb image: (**a**) original, (**b**) Histogram, (**c**) ground-truth image, (**d**) Shannon method (threshold value T=158), (**e**) Albuquerque’s method (entropic parameter q=0.8 and T=158), (**f**) Lin & Ou’s method (q=0.8 and T=78), (**g**) Nie et al.’s method (entropic parameter r=1.297 and T=82), and (**h**) the proposed method (r=0.84 and T=99).

**Figure 12:**
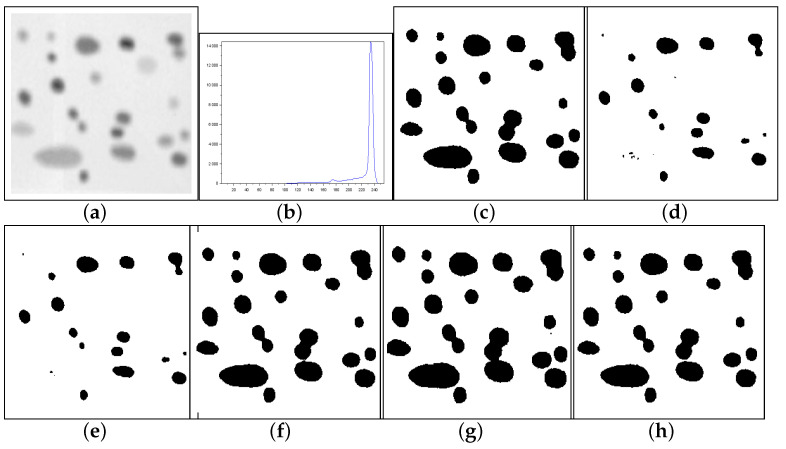
Thresholding results of Cell image: (**a**) original, (**b**) Histogram, (**c**) ground-truth image, (**d**) Shannon method (threshold value T=172), (**e**) Albuquerque’s method (entropic parameter q=0.7 and T=171), (**f**) Lin & Ou’s method (q=0.7 and T=214), (**g**) Nie et al.’s method (entropic parameter r=1.2 and T=222), and (**h**) the proposed method (r=1.25 and T=213).

Figure 10, Figure 11 and Figure 12 form a set of three nondestructive testing (NDT) images, which are further tested. Table 4 presents quantitative performance metrics for the methods on these images. The last column of Table 4 shows that our proposal is the most efficient among the approaches considered. Below, we offer more details about the quantitative results reported in Table 4.

- Figure 10—Gear image: Although the Lin & Ou’s and Nie et al.’s methods have obtained a good visual approximation of the ground truth, the result of our technique matches the ground-truth image with JS=100%, according to Table 4. For this image, the computational experiments have shown that Albuquerque’s method is very sensitive to the variation of parameter *q*. Since this image was not applied in Lin & Ou’s paper, the *q* values were determined by trial-and-error to optimize performance. This allowed Albuquerque’s result to be more than 94% accurate according to JS. The image histogram suggests how easy it would be for the method to separate the regions of the image. As shown in Figure 10b below, there is a sharp peak at the beginning of the histogram. It can be considered as the strong correlations of the pixels in the foreground. Thus, as performed in Lin & Ou’s experiments, the alternative form, (11), was necessary for this image. The same occurred for our method, which used an alternative form to Equation (12) given by Equation (13). This happens due to a weaker long-range correlation in the background composed of the lightest area of the image.
- Figure 11—Pcb image: In this example, Nie et al.’s technique surpasses Lin & Ou’s, having more than 90% of similarity with the ground truth image. The image obtained with Lin & Ou’s method was generated with the alternative form (11). Even so, our method is more efficient than the others, making the accuracy of 99.33%, as shown in Table 4.
- Figure 12—Cell image: Lin & Ou’s method is more effective than Nie et al.’s method, in this case. However, it still does not overcome our result, which is 100% accurate (see Table 4).

The presented set of results proves the superiority of our method against its counterparts in the application to IR and NDT images.

### 6.3. Selection of Entropic Parameter

The procedure used to set the entropic parameter values in Section 6.1 and Section 6.2, as explained in the introduction of Section 6, is ad hoc. In this section, we show a systematic procedure summarized on the following iterative Algorithm 1 that incorporates the proposed thresholding method (Section 4) and applies simulated annealing (SA) to update the *r* value in each iteration, besides the uniformity measure UM, defined in expression (34), and an error measure to compare the binarized image with the ground truth (if available).
**Algorithm 1** Pseudo-code for the thresholding approach proposed based on SA.1:Input: grayscale image *I* with *N* pixels; image intensity range: $\left[0,L\right]$; $T_{initial}$$T_{min}$; α; $markov_{-}length$; $Max_{iter}$; ground truth segmentation $I_{gt}$; Reference error $E_{ref}$; Reference uniformity measure $UM_{ref}$; *d*; $\hat{r}$; fix_par;2:Calculate image histogram $h=h\left(t\right)$;3:**Proposal 1**4:Initial guess of entropic parameter: $\hat{r}$;5:$T_{0}\leftarrow T_{initial}$;6:Objective function:(35)$$F_{1}\left(t,\hat{r}\right)=min\;\left[S^{A}\left(t\right),S_{\hat{r}}^{B}\left(t\right)\right];\;0\leq t\leq L;$$7:Solve the optimization problem:(36)$$t_{opt}\left(\hat{r}\right)=\arg\max_{0\leq t\leq L}\left\{F_{1}\left(t,\hat{r}\right)\right\};$$8:Binarization of input image: $Binary\left(\hat{r}\right)=Thresholding\left(I,t_{opt}\left(\hat{r}\right)\right)$;9:**if** fix_par==0 **then**10:    go to line 44;11:**end if**12:**if** ground truth $I_{gt}$ is available **then**13:    Calculate Misclassification Error: $E\left(0\right)=Error\left(I_{gt},Binary\left(\hat{r}\right)\right)$;14:    If $E\left(0\right)\leq E_{ref}$ then go to Line 44;15:**else**16:    Calculate $UM\left(j\right)=UM\left(t_{opt}\left(\hat{r}\right)\right)$;17:    If $UM\left(j\right)\geq UM_{ref}$ then go to Line 44;18:**end if**19:i←0;20:**while** $T_{i}>T_{min}$ and $i<Max_{iter}$ **do**21:    **for** j=1, j++, while $j<markov_{-}length$; **do**22:        Update $r_{j}\leftarrow \hat{r}+rand\left(-d,d\right)$;23:        Solve the optimization problem:(37)$$t_{opt}\left(r_{j}\right)=\arg\max_{0\leq t\leq L}\left\{F_{1}\left(t,r_{j}\right)\right\};$$24:        Binarization of input image: $Binary\left(r_{j}\right)=Thresholding\left(I,t_{opt}\left(r_{j}\right)\right)$;25:        **if** ground truth $I_{gt}$ is available **then**26:           Calculate Misclassification Error: $E\left(j\right)=Error\left(I_{gt},Binary\left(r_{j}\right)\right)$;27:           If $E\left(j\right)\leq E_{ref}$ then go to Line 44;28:           Calculate difference: $\Delta E=E\left(j\right)-E\left(j-1\right)$;29:           Acceptance criterion: If ΔE≤0 then $\hat{r}\leftarrow r_{j}$ else $prob=\exp\left(\Delta E/T\right)$;30:           If ΔE>0 and $rand\left(0,1\right)<prob$ then $\hat{r}\leftarrow r_{j}$;31:        **else**32:           Calculate $UM\left(j\right)=UM\left(t_{opt}\left(r_{j}\right)\right)$;33:           If $UM\left(j\right)\geq UM_{ref}$ then go to Line 44;34:           Calculate difference: $\Delta UM=UM\left(j\right)-UM\left(j-1\right)$;35:           Acceptance criterion: If ΔUM≥0 then $\hat{r}\leftarrow r_{j}$ else $prob=\exp\left(\Delta UM/T\right)$;36:           If ΔUM<0 and $rand\left(0,1\right)<prob$ then $\hat{r}\leftarrow r_{j}$;37:        **end if**38:    **end for**39:    i←i+1;40:    $T_{i}\leftarrow T_{i-1}\cdot \alpha$;41:**end while**42:**Proposal 2**43:Repeat steps 2–34 with the Objective Function:(38)$$F_{2}\left(t,r\right)=min\;\left[S_{r}^{A}\left(t\right),S^{B}\left(t\right)\right];\;0\leq t\leq L;$$44:Output: Threshold $t_{opt}$ and parameter $\hat{r}$.

In Algorithm 1, we include the flag fix_par to add the possibility of using the algorithm just to obtain the segmentation but without optimizing the entropic parameter. Expressions (35) and (38) implement the objective functions of our proposal. Lines 16–35 incorporate the SA procedure to seek the best *r* value. The computational complexity of Algorithm 1 is defined by the following steps:

1. Computational complexity of Proposal 1:
  (a) Line 2: $O\left(N\right)$;
  (b) Line 7: $O\left(L^{2}\right)$;
  (c) Line 8: $O\left(N\right)$;
  (d) Line 11: $O\left(N\right)$;
  (e) Line 14: $O\left(N\right)$;
  (f) Line 20: $O\left(L^{2}\right)$;
  (g) Line 21: $O\left(N\right)$;
  (h) Line 23: $O\left(N\right)$;
  (i) Line 29: $O\left(N\right)$;
  (j) Line 37: Analogous to Proposal 1.

Consequently, based on items (a)–(j) above, the computational complexity of Algorithm 1 is

$$Comp\left(\mathrm{Algorithm}\;1\right)$$

(39) $$=O\left(Max_{iter}\cdot markov_{-}length\cdot \left[\max\left\{N,L^{2}\right\}\right]\right).$$

which is not a drawback of our proposal, as will be shown in Section 6.5. Appendix A shows a variation of Algorithm 1 obtained by using just the Masi entropy. This algorithm is used to perform ablation analysis in the remaining text.

Besides computational complexity analysis, other important points regarding Algorithm 1 must be considered with respect to the behavior of our approach when varying the entropic parameter. Hence, we propose the following strategy. We selected the image that provided the best value for ME in our initial experiments, the panzer image in Figure 6. We set the following parameterization of Table 5 for Algorithm 1 unless stated otherwise. Then, we establish the steps listed below.

**Figure 13:**
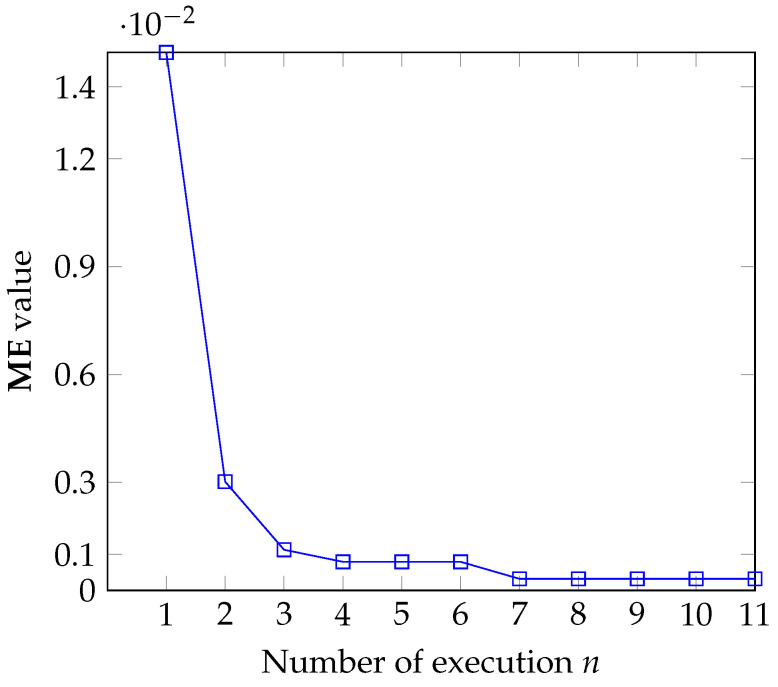
Results of convergence test: ME versus number of executions.

**Figure 14:**
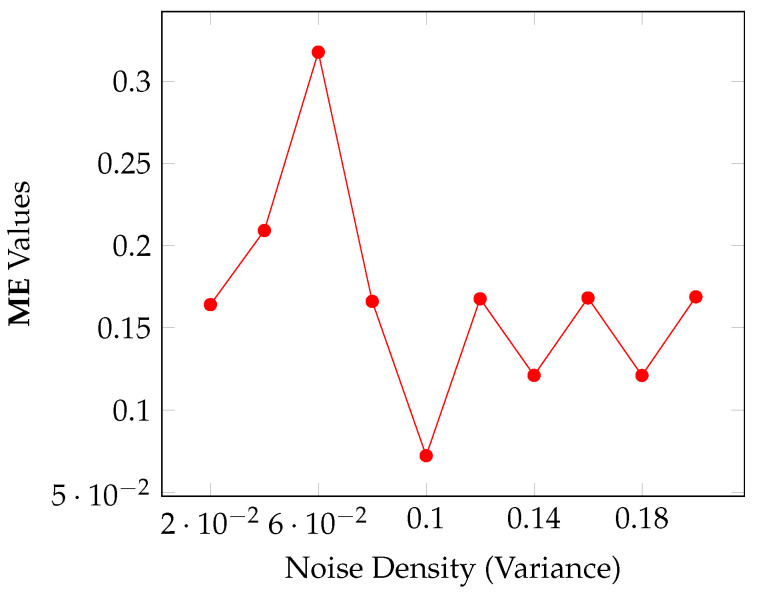
ME behavior against variance in noisy images for Algorithm 1.

1. Sensitivity test: Another important point is the sensitivity to entropic parameter perturbation for the proposed method. We perturb the $\hat{r}$ returned by Algorithm 1 for the panzer image to generate the sequence $r_{k}=\hat{r}+k\Delta r$, where k=−3,−2,−1,0,1,2,3 and Δr=0.01. Then, we compute the sequence $\Delta ME\left(k\right)=|ME\left(k\right)-ME_{ref}|$, where ME(k) is the misclassified error of the segmentation obtained with Algorithm 1 with $\hat{r}=r_{k}$ and fix_par=0 in the input of Algorithm 1, since in this case the *r* value is fixed. Additionally, $ME_{ref}=0.000518798828125$, which is the reference ME value for the panzer image. The other input parameters are maintained as specified in Table 5. Table 6 shows the data obtained in this procedure. Although the sensitivity *S* increases when $r_{k}$ deviates from the reference value $r_{0}$, we notice that 0≤ΔME(k)≤0.00823975, which indicates the stability of Algorithm 1 with respect to parameter variation.
2. Convergence test: Algorithm 1 is executed *n* times with the parameterization of Table 5. The first execution uses $\hat{r}$ as specified in that table. Next, each time it is executed, a parameter $\hat{r}$ is returned, and this parameter is then used again as the initial guess of *r* for the next execution. This process repeats for a number *n* of times or until the algorithm converges, that is, the value of **ME** is equal to 0 or is fixed at a value close to it. The goal here is to check the efficiency of Algorithm 1 to achieve a near-optimum *r* value; that means it is expected that Algorithm 1 converges within a small *n*.
  Figure 13 shows the behavior of Algorithm 1 for the case n=11. From the seventh execution of that Algorithm, the **ME** value converges to 0.0003204345703125, indicating the capability of the SA algorithm to output a near-optimum value for the entropic parameter *r*.
3. Noise sensitivity: Corrupted versions of the panzer reference image were created using a Gaussian distribution to verify the behavior of Algorithm 1 against noise. In this case, 10 noise levels were generated with the standard deviation σ ranging from [0.02,0.04,…,2.0], with increments of 0.02. Furthermore, because the noise pattern must be different each time it is generated, for each noise level we generate 20 noisy images, leading to a total of 200 images. An average of the ME values was calculated for each σ. The *r* and $t_{opt}$ values used for thresholding each image were provided by Algorithm 1 with the parameterization of Table 5.
  The ME results are shown in Figure 14, which shows an unexpected behavior since the ME for $\sigma =6\cdot 10^{-2}$ is higher than the ME for σ=0.18. Despite this, the ME values fall below 0.17 in most cases, which suggests low sensitivity against noise.

### 6.4. Ablation Analysis

We shall notice that Algorithm 1 has a stochastic process inside the *WHILE* loop that starts in line 17 (analogous to Algorithm A1, in line 16) due to the utilization of function *rand*. Consequently, different executions of each algorithm with the same input may produce different results for $\hat{r}$. Steered by this fact, in this section, we kept the panzer image as input and generate twenty $\hat{r}$ values using twenty executions of Algorithm 1 and twenty more $\hat{r}$ values were generated using twenty executions of Algorithm A1, yielding the sequence ${\hat{r}}_{1}^{j},{\hat{r}}_{2}^{j},\ldots ,{\hat{r}}_{20}^{j}$), j=1,2 of values for $\hat{r}$. The parameterization is given in Table 5 for Algorithms 1 and A1. We then collected the ME and UM values, obtained using ${\hat{r}}_{i}^{j}$, i=1,…,20, j=1,2 and plotted the corresponding curves against $\hat{r}$. This experiment aims to verify the influence of the stochastic process on the output.

**Figure 15:**
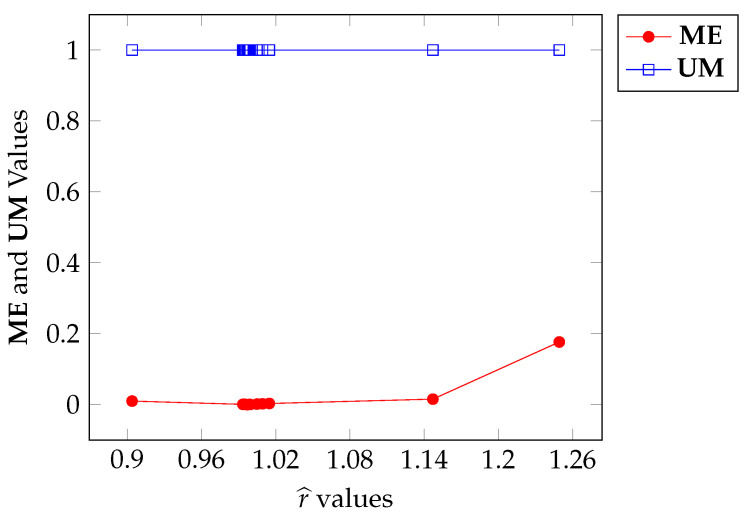
ME and UM variation with respect to $\hat{r}$ calculated through Algorithm 1.

**Figure 16:**
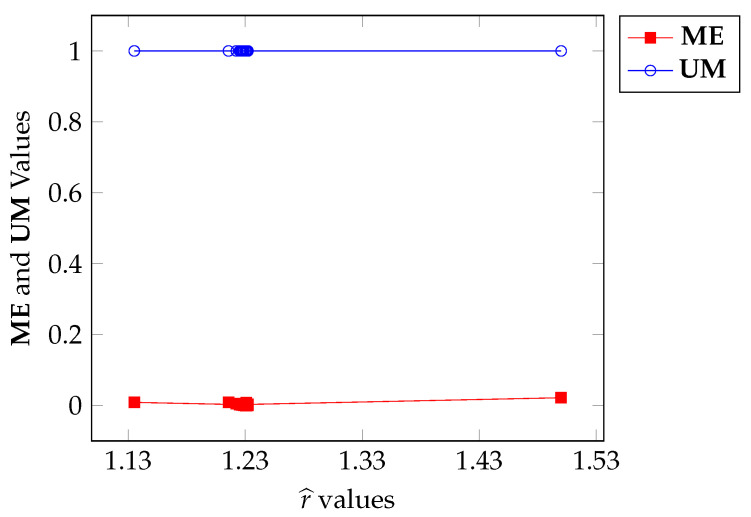
ME and UM variation with respect to $\hat{r}$ calculated through Algorithm A1.

Figure 15 and Figure 16 show the plots of ME versus $\hat{r}$ for Algorithms 1 and A1, respectively. Firstly, the range for $\hat{r}$ for Algorithm 1 is [0.9,1.26] while the range for the other algorithm is [1.13,1.53]. Although both algorithms start with $\hat{r}=1.5$, their objective functions are different, which justifies the differences in the ranges.

In both cases, the ME values are very close to zero, if not reaching it. Algorithm 1, in this case, sets the ME value to 0 in 4 instances, within the range [0.9968,0.9975] of $\hat{r}$. On the other hand, Algorithm A1 sets the ME value to 0 in only 2 instances, in the range [1.230,1.232] of $\hat{r}$. In other words, the simulated annealing method leads to a higher convergence rate for Algorithm 1 with a slight advantage over Algorithm A1.

Note that the values obtained for $\hat{r}$ in the original experiment (see Figure 6) are in the ranges provided by simulated annealing, as observed in Figure 15. It is important to note that more than one ME value was set to 0 in both cases. This demonstrates the existence of several local minima for ME, i.e., several local maxima for functions in expressions (35) and (A1). In this way, the experiments of Section 6.1 are validated by the latter (Algorithms 1 and A1).

Regarding the UM values, these are concentrated in values very close to 1 for both Algorithms 1 and A1. This corroborates all the observations made for the ME measure made above.

### 6.5. Performance on BSDS500 Images

This section has a two-fold proposal. Firstly, demonstrates the applicability of our proposal out of the context of IR and NDT images. Secondly, compare the performance of Algorithm 1 against counterpart methods based on entropy concepts, as well as the support vector machine (SVM) algorithm, which is a known machine learning approach.

The comparison with entropy-based methods is executed by considering the competing methods reported in the first column of Table 7: Albuquerque [3], based on the Tsallis entropy; Nie et al. [6], based on Masi entropy; Lin & Ou [5], based on Shannon and Tsallis entropies; Deng [37], based on Tsalis entropy; Manda & Hyun [36], based on Sine entropy; Elaraby & Moratal [35], based on Hill entropy; Proposals 1 and 2 of [38], based on Tsallis and Masi entropies. The performance of each technique is reported in Table 7. It is achieved by computing the mean and standard deviation (Std) of the ME over the segmentations of the set BSDS500-50, described in the introduction of Section 6. The BSDS500-50 images were converted to grayscale format since the considered methods do not work with RGB data. We observe that our approach is the most outstanding one, as its mean and standard deviation are the smallest. In all these experiments the parmeterization of Algorithm 1 is specified in Table 5.

Now, we compare Algorithm 1’s efficiency with the segmentation obtained by a SVM approach. The SVM is a machine learning technique that, in our application, seeks the classification hypersurface that maximizes the separation margin between foreground and background pixels. Hence, our SVM approach works in the pixel space. Consequently, we must separate two disjoint subsets: the training one ($D_{tr}$), which in this case is composed of a pool of pixels used to compute the SVM hypersurface, and the test set ($D_{te}$) to evaluate the segmentation (inference) obtained.

**Figure 17:**
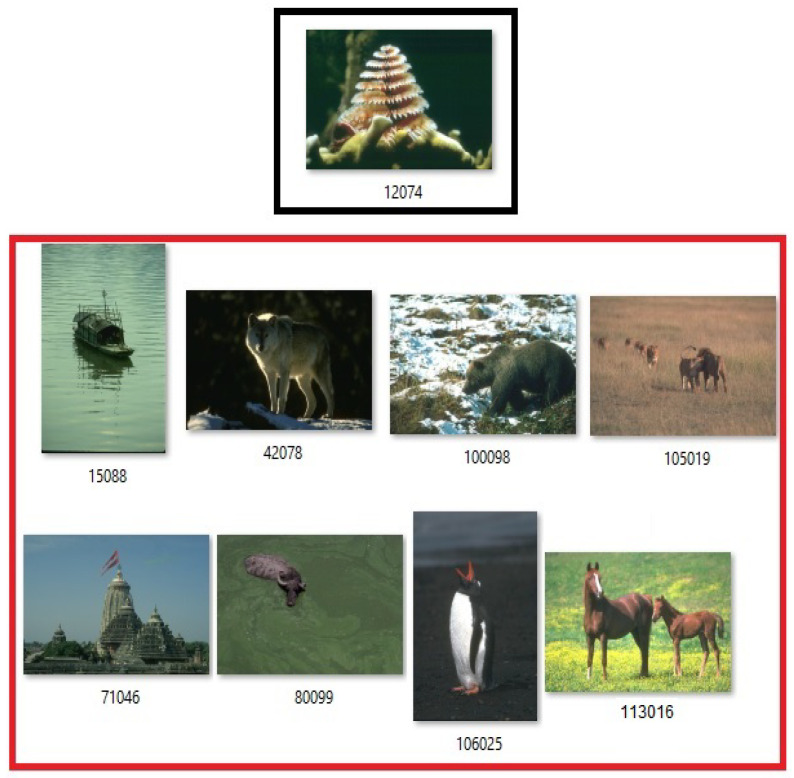
Images used for SVM training (black rectangle) and inference (red rectangle).

As usual in the machine learning area, the selection of the $D_{tr}$ is critical to obtaining a good performance over the test set $D_{te}$. Since the BSDS500-50 images present different contents and characteristics, as observed in Figure 2, selecting images to compose the pixels in the training set is far from trivial. Hence, to offer a meaningful comparison between our approach and SVM, we randomly select the image *12074.jpg*, shown in Figure 17, to generate the $D_{tr}$. Hence, we generate a list of training points $D_{tr}=\left\{\left(R_{i},G_{i},B_{i};l_{i}\right),\;1\leq i\leq N_{tr}\right\}$ where the first three components are the color channels of each pixel, $l_{i}=1$ for the foreground and $l_{i}=-1$ for the background, and $N_{tr}=481\cdot 321=154401$. The SVM used is the non-separable one, with constant C=1 and the RBF kernel.

**Figure 18:**
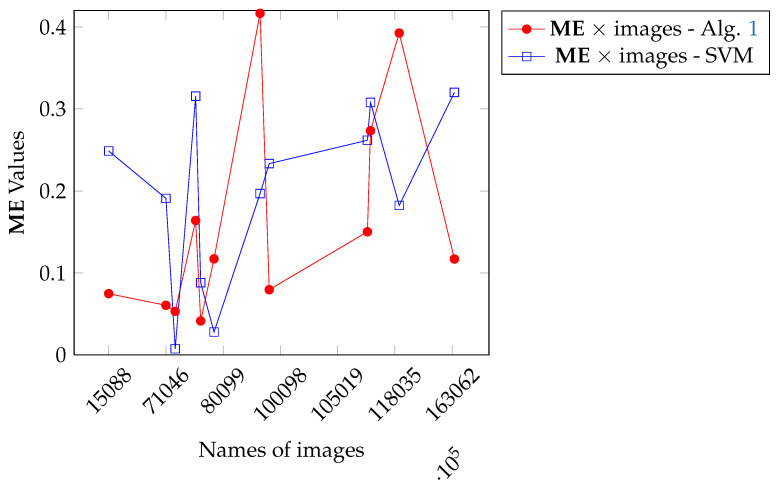
Comparison between Algorithm 1 and SVM results.

Then, we perform inference over the $D_{te}$ set, but processing one image at a time. Next, we selected the test images for which the SVM achieved an ME larger than 0.3% and performed its segmentation with Algorithm 1 for comparison. Figure 17 pictures the training image and the test ones. The ME values for both SVM and Algorithm 1 over selected images can be analyzed in Figure 18.

Figure 18 shows that in most cases, our method outperforms that of SVM, except in four instances: 80099, 163062, 271008, and 299091. Obviously, if we change the $D_{tr}$ or the SVM parametrization, or use a more powerful machine learning method (see Section 7), we can obtain a different result. Despite this, the results presented in Figure 18 indicate that Algorithm 1 is competitive with respect to a traditional machine learning approach.

## 7. Discussion

The long-range correlations of an image can be captured by the Tsallis and Masi entropies. In particular, IR and NDT images show local long-range correlation instead of global long-range correlation. In this work, the image segmentation technique based on Tsallis entropy and long-range correlation proposed by Lin & Ou was improved. The combination of Masi and Shannon entropies not only outperforms that method but has also shown itself to be competitive against works that apply only Masi or only Tsallis models. Misclassification error, Jaccard similarity, misclassified pixels, relative foreground area error, and *F*—measure have proven the effectiveness of our method.

Regarding the comparison between Algorithm 1 and deep neural networks for image segmentation, like U-Net and others [65], some points must be contemplated:

1. The proposed approach does not rely on training stages. It is advantageous to avoid the computational complexity of such stages, which is a significant challenge for deep learning models.
2. The training process of machine learning models allows the incorporation of prior information into the segmentation model. It is an advantage of that stage despite its computational cost. In the case of Algorithm 1, given a dataset *D* composed of images with similar contents, we can take an image *I* from *D*, generate its ground truth $I_{gt}$, and apply Algorithm 1 with *I* and $I_{gt}$ as input. The returned Masi parameter $\hat{r}$ can be used as an initial guess of *r* for the remaining images from *D*. This strategy *simulates* a training process and could reduce the complexity of Algorithm 1, considering the low sensitivity of our proposal with respect to *r* verified in Section 6.3.
3. The comparison between SVM and Algorithm 1 shows that the latter is competitive regarding the former. The machine learning performance could be improved by using a deep neural network with sufficient degrees of freedom. However, considering the limited number of annotated images in the BSDS500-50 dataset, we should fine-tune a pre-trained neural network, a process that also has its drawbacks [66].
4. The usual strategy for deep learning segmentation works in the image space; that means the model input is an image *I* instead of the pixel channels $\left(R,G,B\right)$ as performed in Section 6.5. However, in this case, it is not expected that BSDS500-50 will be enough for training, considering the diversity of patterns observed in Figure 2 and Figure 17. In fact, we need a large training dataset $D_{tr}$ even when using pre-trained neural networks. In summary, the performance verified in Section 6.5 for Algorithm 1 indicates that it is a suitable choice for applications with limited training data.
5. Besides issues involving the training stage, deep neural networks have also several parameters to be set in advance (hyperparameters). There is no systematic theory for hyperparameter optimization. The state-of-the-art approaches, like Bayesian, evolutionary algorithms, and grid search [67], depend on computationally intensive tests, which is another issue for deep learning approaches. According to Table 5, the *hyperparameters* space of Algorithm 1 has dimension 9, since *L* and fix_par are defined by the application requirements. Such a dimension is much smaller compared with deep learning approaches.

With respect to issues related to our proposal, we shall return to Algorithm 1 and notice that there is no guarantee that the criterion in line 24 (or 30) will eventually be satisfied. In such a case, the parameterization of the algorithm must be reconsidered, for instance, by changing the initial guess $\hat{r}$, increasing the computational cost. The incorporation of prior knowledge in the segmentation process, a natural procedure for machine learning approaches during training, is not considered in Algorithm 1, which is a disadvantage compared to deep learning models, for instance, despite the computational cost to train the model. In addition, the proposed method deals only with bi-level thresholding. Its extension to the multilevel case will be addressed in further work.   

## Figures and Tables

**Table 1:** Comparison between the techniques. Ideal values: ME=0, JS=100%, RAE=0, $F_{\alpha }=1$. In **black**, the best results are highlighted.

Images		Thresholding Methods				
		Shannon	Albuquerque	Lin & Ou	Nie et al.	Our Method
000280	Threshold	91	91	169	173	170
	Misclassified pixels	4900	4900	6	6	4
	**ME**	0.0638021	0.0638021	$7.81\times 10^{-05}$	$7.81\times 10^{-05}$	$5.21\times 10^{-05}$
	JS (%)	3.94	3.94	97.06	97.02	98.01
	RAE	0.960596	0.960596	0	0.0298507	0.0199005
	$F_{\alpha }$	0.057964	0.057964	0.985075	0.989848	0.993277
Airplane	Threshold	117	117	117	121	123
	Misclassified pixels	44	44	44	8	4
	**ME**	0.000671387	0.000671387	0.000671387	0.00012207	$6.10\times 10^{-05}$
	JS (%)	93.05	93.05	93.05	98.66	99.32
	RAE	0.0695103	0.0695103	0.0695103	0.0134003	0.00679117
	$F_{\alpha }$	0.952561	0.952561	0.952561	0.991026	0.997726
Tank	Threshold	140	140	220	194	191
	Misclassified pixels	390	390	255	21	9
	ME	0.0133929	0.013929	0.00875687	0.000721154	0.000309066
	JS (%)	59.29	59.29	55.11	96.30	98.44
	RAE	0.407098	0.407098	0.448944	0.0369718	0.0155979
	$F_{\alpha }$	0.68599	0.68599	0.786432	0.987365	0.989547
Panzer	Threshold	154	157	153	174	174
	Misclassified pixels	574	426	642	0	0
	**ME**	0.00875854	0.00650024	0.00979614	0	0
	JS (%)	75.23	80.36	73.08	100.00	100.00
	RAE	0.247734	0.196404	0.269182	0	0
	$F_{\alpha }$	0.819978	0.859891	0.802856	1	1
Car	Threshold	81	81	80	89	90
	Misclassified pixels	492	492	581	55	11
	**ME**	0.00750732	0.00750732	0.00886536	0.000839233	0.000167847
	JS (%)	60.17	60.16	56.12	93.11	98.54
	RAE	0.398381	0.398381	0.438822	0.0689223	0.0145889
	$F_{\alpha }$	0.693744	0.693744	0.657328	0.952971	0.990227
Sailboat	Threshold	155	155	141	155	193
	Misclassified pixels	1295	1295	2447	1295	98
	**ME**	0.0197601	0.0197601	0.0373383	0.0197601	0.00149536
	JS (%)	41.46	41.46	27.26	41.46	90.35
	RAE	0.585443	0.585443	0.727408	0.585443	0.0965517
	$F_{\alpha }$	0.515072	0.515072	0.359843	0.515072	0.933492

**Table 2:** The thresholding accuracy (JS, Equation (12)) of methods. Ideal value: JS = 100%. In **black**, the best results are highlighted.

Images	Albuquerque	Lin & Ou	Nie et al.	Our
	(%)	(%)	(%)	(%)
000280	3.94	97.06	96.02	97.51
000300	4.25	94.47	94.04	95.87
000320	3.75	90.05	94.91	94.93
000340	4.85	95.02	94.59	95.02
000360	4.26	91.21	92.08	92.08
000380	5.98	89.78	90.38	92.36
000400	4.23	98.20	100.00	100.00
000420	3.96	97.51	98.98	99.49
000440	3.87	93.67	97.61	97.61
000460	5.12	94.05	96.86	98.05
**Average**	4.42	94.10	95.55	96.29

**Table 3:** Comparison between the techniques concerning misclassified pixels and ME. Ideal value: ME=0. In **black**, the best results are highlighted.

Images		Thresholding Methods			
		Albuquerque	Lin & Ou	Nie et al.	Our Method
000280	Threshold	91	169	173	170
	Misclassified pixels	4900	6	6	4
	ME	0.0638021	$7.81\times 10^{-05}$	$7.81\times 10^{-05}$	$5.21\times 10^{-05}$
000300	Threshold	93	178	177	174
	Misclassified pixels	4895	12	13	9
	ME	0.063737	0.00015625	0.000169271	0.000117188
000320	Threshold	91	185	171	170
	Misclassified pixels	5413	21	11	11
	ME	0.0704818	0.000273437	0.000143229	0.000143229
000340	Threshold	97	177	176	177
	Misclassified pixels	4339	11	12	11
	ME	0.0564974	0.000143229	0.00015625	0.000143229
000360	Threshold	90	179	175	175
	Misclassified pixels	5355	21	19	19
	ME	0.0697266	0.000273437	0.000247396	0.000247396
000380	Threshold	98	178	170	174
	Misclassified pixels	4309	28	28	21
	ME	0.0561068	0.000364583	0.000364583	0.000273437
000400	Threshold	98	179	177	177
	Misclassified pixels	5033	4	0	0
	ME	0.0655339	$5.21\times 10^{-05}$	0	0
000420	Threshold	96	173	176	175
	Misclassified pixels	4756	5	2	1
	ME	0.0619271	$6.51\times 10^{-05}$	$2.60\times 10^{-05}$	$1.30\times 10^{-05}$
000440	Threshold	94	175	178	178
	Misclassified pixels	5142	14	5	5
	ME	0.0669531	0.000182292	$6.51\times 10^{-05}$	$6.51\times 10^{-05}$
000460	Threshold	96	192	182	181
	Misclassified pixels	4654	15	8	5
	ME	0.060599	0.000195313	0.000104167	$6.51\times 10^{-05}$

**Table 4:** Comparison between the—NDT images. Ideal values: ME=0, JS=100%, RAE=0, $F_{\alpha }=1$. In **black**, the best results are highlighted.

Images		Thresholding Methods				
		Shannon	Albuquerque	Lin & Ou	Nie et al.	Our Method
Gear	Threshold	185	128	87	75	**80**
	Misclassified pixels	8200	1553	188	139	**0**
	ME	0.125122	0.0236969	0.00286865	0.00212097	**0**
	JS (%)	73.05	94.90	99.38	99.55	**100.00**
	RAE	0.269506	0.0510419	0.00617893	0.00454769	**0**
	$F_{\alpha }$	0.890489	0.982387	0.997932	0.996964	**1**
Pcb	Threshold	158	158	78	82	**99**
	Misclassified pixels	13852	13852	2808	2166	**145**
	ME	0.211365	0.211365	0.0428467	0.0330505	**0.00221252**
	JS (%)	36.01	36.01	88.52	90.90	**99.33**
	RAE	0.639904	0.639904	0.114823	0.0909587	**0.00669839**
	$F_{\alpha }$	0.628004	0.628004	0.920405	0.937465	**0.997757**
Cell	Threshold	172	171	214	222	**213**
	Misclassified pixels	7276	7375	271	2634	**0**
	ME	0.111023	0.112534	0.00413513	0.0401917	**0**
	JS (%)	88.26	88.12	99.50	95.19	**100.00**
	RAE	0.117376	0.118783	0.00495312	0.0481421	**0**
	$F_{\alpha }$	0.918563	0.917547	0.998343	0.983421	**1**

**Table 5:** Input parameters used in the executions of Algorithm 1.

Parameter	Value
$\hat{r}$	1.5
*d*	0.1
*L*	255
$T_{initial}$	100.0
$T_{min}$	$10^{-3}$
α	0.99
markov_length	20
max_iter	100
$E_{ref}$	0.0
$UM_{ref}$	1.0
fix_par	0 or 1

**Table 6:** Sensitivity test of the r parameter—panzer image.

*k*	ME Values	Sensitivity (*S*)
−3	0.008758544921875	0.00823975
−2	0.0035400390625	0.00302124
−1	0.0013885498046875	0.00086975
0	0.000518798828125	0
1	0.0021820068359375	0.00166321
2	0.0035247802734375	0.00300598
3	0.0050811767578125	0.00456238

**Table 7:** Mean of ME values and standard deviations (Adapted from [38]).

Thresholding Methods	ME Mean: μ	Std Deviat.: σ	[μ−σ, μ+σ]
Albuquerque [3]	0.32	0.24	[0.08, 0.56]
Nie et al. [6]	0.31	0.25	[0.06, 0.56]
Lin & Ou [5]	0.38	0.25	[0.13, 0.63]
Deng [37]	0.49	0.26	[0.23, 0.75]
Manda & Hyun [36]	0.48	0.25	[0.23, 0.73]
Elaraby & Moratal [35]	0.27	0.19	[0.08, 0.46]
Proposal 1 of [38]	0.31	0.22	[0.09, 0.53]
Proposal 2 of [38]	0.44	0.26	[0.18, 0.70]
Our Algorithm 1	0.005	0.0282	[−0.0232, 0.0332]

## Data Availability

Image data used in this research can be found at https://drive.google.com/file/d/1LzQNCjK4YeTzqGf-hYQ7Q_osi2Ery9m8/view?usp=sharing (accessed on 15 September 2025).

## Abbreviations

    The following abbreviations are used in this manuscript:

$F_{\alpha }$	F-measure
IR	Infrared Images
JS	Jaccard Similarity
ME	Misclassification Error
NDT	Nondestructive Images
PDEs	Partial Differential Equations
RAE	Relative Foreground Area Error

## Appendix A. Masi Thresholding Algorithm

This section presents a variation of Algorithm 1, obtained by using just the Masi entropy, aiming to allow ablation analysis of Algorithm 1. The new procedure incorporates the same SA methodology of Algorithm 1 and the possibility of using it just to compute the threshold $t_{opt}$ given a fixed *r* value (set fix_par=1).

**Algorithm A1** Pseudo-code for Masi thresholding based on SA.
1: Input: grayscale image *I* with *N* pixels; image intensity range: $\left[0,L\right]$; $T_{initial}$$T_{min}$; α; $markov_{-}length$; $max_{iter}$; groud truth segmentation $I_{gt}$; Reference error $E_{ref}$; Reference uniformity measure $UM_{ref}$; *d*; $fix\text{\_}par\in \left\{0,1\right\}$; 2: Calculate image histogram $h=h\left(t\right)$; 3: Initialization of entropic parameter: $\hat{r}$; 4: $T_{0}\leftarrow T_{initial}$; 5: Objective function: (A1) $$F_{3}\left(t,\hat{r}\right)=S_{\hat{r}}^{A+B}\left(t\right);\;0\leq t\leq L;$$ 6: Solve the optimization problem: (A2) $$t_{opt}\left(\hat{r}\right)=\arg\max_{0\leq t\leq L}\left\{F_{3}\left(t,\hat{r}\right)\right\};$$ 7: Binarization of input image: $Binary\left(\hat{r}\right)=Thresholding\left(I,t_{opt}\left(\hat{r}\right)\right)$; 8: **if** fix_par==0 **then** 9: go to line 41; 10: **end if** 11: **if** ground truth $I_{gt}$ is available **then** 12: Calculate Misclassification Error: $E\left(0\right)=Error\left(I_{gt},Binary\left(\hat{r}\right)\right)$; 13: If $E\left(0\right)\leq E_{ref}$ then go to Line 41; 14: **else** 15: Calculate $UM\left(j\right)=UM\left(t_{opt}\left(\hat{r}\right)\right)$; 16: If $UM\left(j\right)\geq UM_{ref}$ then go to Line 41; 17: **end if** 18: i←0; 19: **while** $T_{i}>T_{min}$ and $i<Max_{iter}$ **do** 20: **for** j=1, j++, while $j<markov_{-}length$; **do** 21: Update $r_{j}\leftarrow \hat{r}+rand\left(-d,d\right)$; 22: Solve the optimization problem: (A3) $$t_{opt}\left(r_{j}\right)=\arg\max_{0\leq t\leq L}\left\{F_{3}\left(t,r_{j}\right)\right\};$$ 23: Binarization of input image: $Binary\left(r_{j}\right)=Thresholding\left(I,t_{opt}\left(r_{j}\right)\right)$; 24: **if** ground truth $I_{gt}$ is available **then** 25: Calculate Misclassification Error: $E\left(j\right)=Error\left(I_{gt},Binary\left(r_{j}\right)\right)$; 26: If $E\left(j\right)\leq E_{ref}$ then go to Line 41; 27: Calculate difference: $\Delta E=E\left(j\right)-E\left(j-1\right)$; 28: Acceptance criterion: If ΔE≤0 then $\hat{r}\leftarrow r_{j}$ else $prob=\exp\left(\Delta E/T\right)$; 29: If ΔE>0 and $rand\left(0,1\right)<prob$ then $\hat{r}\leftarrow r_{j}$; 30: **else** 31: Calculate $UM\left(j\right)=UM\left(t_{opt}\left(r_{j}\right)\right)$; 32: If $UM\left(j\right)\geq UM_{ref}$ then go to Line 41; 33: Calculate difference: $\Delta UM=UM\left(j\right)-UM\left(j-1\right)$; 34: Acceptance criterion: If ΔUM≥0 then $\hat{r}\leftarrow r_{j}$ else $prob=\exp\left(\Delta UM/T\right)$; 35: If ΔUM<0 and $rand\left(0,1\right)<prob$ then $\hat{r}\leftarrow r_{j}$; 36: **end if** 37: **end for** 38: i←i+1; 39: $T_{i}\leftarrow T_{i-1}\cdot \alpha$; 40: **end while** 41: Output: Threshold $t_{opt}$ and parameter $\hat{r}$.
